# Processing of Biomass Prior to Hydrogen Fermentation and Post-Fermentative Broth Management

**DOI:** 10.3390/molecules27217658

**Published:** 2022-11-07

**Authors:** Zhila Honarmandrad, Karolina Kucharska, Jacek Gębicki

**Affiliations:** Department of Process Engineering and Chemical Technology, Faculty of Chemistry, Gdansk University of Technology, 11/12 Gabriela Narutowicza Street, 80-233 Gdansk, Poland

**Keywords:** detoxification, biohydrogen, green solvents, biomass, lignocellulose

## Abstract

Using bioconversion and simultaneous value-added product generation requires purification of the gaseous and the liquid streams before, during, and after the bioconversion process. The effect of diversified process parameters on the efficiency of biohydrogen generation via biological processes is a broad object of research. Biomass-based raw materials are often applied in investigations regarding biohydrogen generation using dark fermentation and photo fermentation microorganisms. The literature lacks information regarding model mixtures of lignocellulose and starch-based biomass, while the research is carried out based on a single type of raw material. The utilization of lignocellulosic and starch biomasses as the substrates for bioconversion processes requires the decomposition of lignocellulosic polymers into hexoses and pentoses. Among the components of lignocelluloses, mainly lignin is responsible for biomass recalcitrance. The natural carbohydrate-lignin shields must be disrupted to enable lignin removal before biomass hydrolysis and fermentation. The matrix of chemical compounds resulting from this kind of pretreatment may significantly affect the efficiency of biotransformation processes. Therefore, the actual state of knowledge on the factors affecting the culture of dark fermentation and photo fermentation microorganisms and their adaptation to fermentation of hydrolysates obtained from biomass requires to be monitored and a state of the art regarding this topic shall become a contribution to the field of bioconversion processes and the management of liquid streams after fermentation. The future research direction should be recognized as striving to simplification of the procedure, applying the assumptions of the circular economy and the responsible generation of liquid and gas streams that can be used and purified without large energy expenditure. The optimization of pre-treatment steps is crucial for the latter stages of the procedure.

## 1. Introduction

The world energy situation is unstable due to environmental, economic, and geopolitical problems. Greenhouse gas emissions from fossil fuel combustion cause global warming, acid rain, climate change, ozone depletion, and biodiversity damage [1]. Declining fossil fuel reserves, increasing pollution, and climate change in the Earth’s atmosphere have made the production and use of renewable energy sources that are less polluting an inevitable necessity in the present age. Various fossil resources such as natural gas, coal, gasoline, and oil are used as energy sources to produce electricity (20%) and fuel (80%) [2].

Therefore, fuel systems excluding carbon dioxide need to be developed and applied in the future. Since biomass is the fourth largest source of energy after oil, coal, and gas, it should be considered raw material for further processing [3] Biofuel is a type of solid, liquid, or gaseous fuel that is obtained from a wide range of biomass sources, including a variety of crops, agricultural and forest residues, aquatic plants, animal waste, and municipal waste. Although a wide variety of biofuels is described in the literature, bioethanol, biodiesel, and biogas are best known [4].

Based on the criterion of origin secondary biofuels are distinguished as first-, second-, and third-generation biofuels. The first-generation biofuels are generated on basis of food origin biomass, i.e., wheat, barley, corn, rice, potatoes, canola, soybeans, almonds, sunflower, palm, coconut, ground beet, sugarcane, etc [5,6]. The second-generation biofuels are generated based on non-food biomass, i.e., lignocellulosic material, including waste from agriculture and forestry, sewage, municipal and industrial waste, and trees grown specifically for energy production (such as spruce, poplar, or willow). The third-generation biofuels are generated with the application of algae and microalgae as feed to produce biofuels [7]. Bio-fraction of polysugar-based waste materials (starch-type and lignocellulosic-type biomass) requires the application of pre-treatment steps to improve the saccharification efficiency and post-fermentation purification to remove or recover derivatives or value-added products, i.e., chemical bio compounds from the broth [8].

Lignocellulosic biomass (LB) is an abundant and renewable source of carbohydrates, consisting mainly of polysaccharides including cellulose and hemicelluloses, and an aromatic polymer called lignin. Lignocellulosic biomass has great potential as an alternative to fossil fuels for the production of second-generation biofuels and chemicals and biomaterials without compromising global food security and without addressing the food versus energy debate [9,10]. Most published research is conducted on the homogenous type of biomass. Since waste biomass is usually a mixture, an overview of the mixture should be taken into account while planning the pre-treatment procedure.

Starch is an abundant natural renewable polymer and has been used in biomaterial applications due to its properties such as biodegradability, low toxicity, and stability. Starch is composed of glucose monomers that are linked together by α-1,4-glycoside bonds and branched by α-1,6-glycoside bonds [11,12]. Efficient saccharification of starch before fermentation requires the application of amylolytic enzymes. The pre-treatment of both lignocellulosic and starch-based materials leads to the generation of a carbohydrates cocktail which can be introduced to dark fermentation.

Hydrogen is the most important source of renewable energy, recognized as environmentally friendly, and can be converted into electricity by fuel cells. Hydrogen has the highest energy production of any known fuel. Its production is possible in various ways by using petroleum products, coal, gas, algae, and the fermentation of bacteria [13].

The object of interest for this review paper is hydrolysate generation for biohydrogen production via dark fermentation by microorganisms, especially bacteria and yeasts. There are two types of fermentation to produce biohydrogen by bacteria: one is photo fermentation, which requires a light source, and the other is dark fermentation, which does not require light. Many carbon sources are used in these reactions, all of which are supplied by biomass [14]. The matrix for biohydrogen generation is complexed and therefore, a large group of derivatives may be generated during the pre-treatment step. The effect of the derivatives on the fermentation efficiency must be taken into account [1,5,6]. The unfermentable compounds occurring in the pre-treated biomass hydrolysates must be also considered, as these substances may be further present in the post-fermentation broth and may interact as promoting or inhibiting agents [11,13].

To our best knowledge, dark hydrogen fermentation is the most widespread and promising biological method of hydrogen synthesis [15]. It is characterized by the high synthesis efficiency of the gas desired in the energy industry [7]. Up to date, the published results are focused mainly on pure cultures maintained under mesophilic conditions, sometimes moving towards higher temperatures, i.e., *Clostridiaceae*, *Flexibacteraceae, Enterobacter,* and *Klebsiella* [13,15]. Dark fermentation is easy to carry, as it does not require light and therefore issues related to light transmission do not occur [15,16]. The microorganisms able to carry dark fermentation show a temperature optimum ranging from 30 °C to 80 °C. The amount of hydrogen produced during fermentation depends on the value of pH, HRT (hydraulic retention time), and pressure. For optimal hydrogen production, a pH value of 5–6 is recommended, which copes well with the organic acids formulation [16]. Anaerobic bacteria generate biohydrogen via the biotransformation of hexoses, mainly glucose, to pyruvate with simultaneous generation of hydrogen during the regeneration of NADH (nicotinamide adenine dinucleotide). The yield of hydrogen depends on the type of fermentation and activity of ferredoxin oxidoreductase and acetyl-coA. While fermentation leads to acetic and formic acids, the yield of hydrogen may be equal to 4 mol H_2_/mol glucose, and when butyric fermentation occurs—up to 2 mol H_2_/mol glucose [10,13]. The acids generated in dark fermentation may be applied as carbon sources in photo fermentation with sulfur-free *Rhodospirillaceae*, including the *Rhodopseudomonas, Rhodospirillum, Rhodobacter,* and *Rhodobium.* High purity of hydrogen is an advantage of photo fermentation (gas contains 10–20% carbon dioxide admixture). This eliminates the compulsion of the energetically and time-consuming purification process of the obtained gas. For this reason, photo fermentation with the use of nitrogenase arouses considerable interest among researchers and practitioners dealing with the synthesis of biohydrogen [13,14,15,16,17,18]. Amongst the disadvantages of the photo fermentation process is the low efficiency, and the economy of biohydrogen generation should be mentioned. Biotechnologists currently use genetic modifications of microorganisms, metabolic engineering, improvement of the reactor structure, or the use of various deposits for cell immobilization, to improve the hydrogen efficiency [18].

Unfortunately, satisfactory results have still not been achieved, and the production of biohydrogen, especially by photo fermentation, of subsequent dark and photo fermentation, remains a crucial problem as energy production on an industrial scale is considered. To make the production of hydrogen by biological methods economically and ecologically feasible, integrated processes need to be developed [15,18,19]. Each planned and optimized set of unit operations in the range from biomass to biohydrogen must take into account the problems that arise in the area of issues related to the processing of the raw materials, conducting fermentation, and management of post-process streams.

This paper presents the current state of knowledge on the relationship between the applied methods of pre-treatment, the derivatives generated during pre-treatment and decomposition products of raw materials, and the yield of hydrogen obtained in the fermentation process. A review of the currently applied techniques enabling the management of post-fermentation broth. The proposed approach is novel, as it considers mixtures of the raw material and the assumptions of circular economy at the early stages of the procedure.

## 2. State of the Art on the Biomass Recalcitrance

### 2.1. Biomass Recalcitrance and the Pre-Treatment of Raw Materials

In recent years, due to the intensification of the energy crisis and arising of environmental pollution awareness, special attention has been paid to the production of biofuels and biochemical fuels from biomass. Among the “biomass for biofuels” research direction, the fraction of municipal biowaste, containing starch and lignocellulose-based materials, is often applied due to the scale of their occurrence as raw materials and products in the industry [19]. Annually, 10 to 50 billion tons of dry lignocellulose is obtained in the world, which is about half of the global biomass yield [20,21].

Biomass recalcitrance is related to the chemical and physical properties of the plant cell wall. Lignin, hemicelluloses, pectin, ash, and their spatial bonds create physical barriers to protect cellulose from degradation. Factors affecting the enzymatic hydrolysis of cellulose biomass include lignin, hemicelluloses, the contents of the recalcitrant group, cellulose crystallization, degree of polymerization, specific surface area, pore-volume, and particle size [22,23].

**Figure 1:**
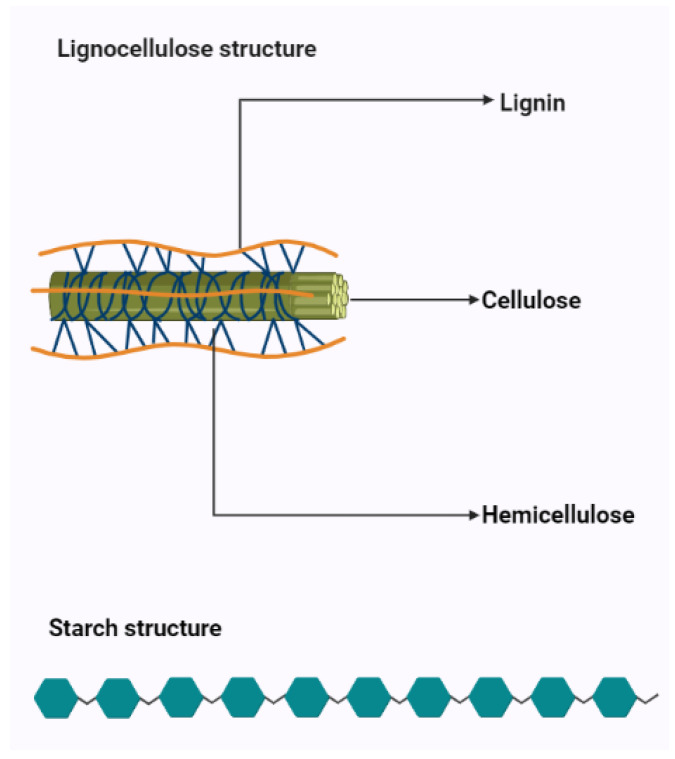
Pictorial diagram of chemical structures of starch and lignocellulose concerning main units in the structure.

Although these factors limit the enzymatic hydrolysis of biomass and have been extensively studied, the molecular mechanisms of biomass resistance are still unclear. Various methods of pre-treatment have been developed over the past few decades [13,15,24,25,26,27,28,29,30]. The biomass recalcitrance occurs due to the presence of diversified monomers in the biomass (Figure 1).

The overall purpose of every pre-treatment is to remove recalcitrant barriers to increase the saccharification of cellulose by altering the chemical composition and physical structures of biomass raw materials. Understanding how chemical compounds and physical structures affect biomass recalcitrance and how they affect the saccharification of lignocelluloses can greatly improve existing pretreatment technologies and promote the development of new pretreatment technologies [21]. The main differences between starch and lignocellulose-based materials and their maintenance have been presented in Table 1, concerning selected criteria, i.e., microbial resistance, availability of monosugars, main units, their interactions, and pre-treatment by-products [20,21,22,23,24,25,26].

The need to explore the issues related to biomass pretreatment to achieve a high degradation of polysugars [31] and to minimize the formation of inhibitors for subsequent fermentation steps is a consequence of the objectives presented for biomass in Table 1. Due to chemical properties, it is obvious that the main source of monosugars, i.e., glucose, is strongly correlated with the starch and cellulose content and availability [25,30].

Starch can be almost completely liquefied while an amylolytic enzyme cocktail is applied [8,32]. However, remnant protein, fibers, fats, and their secondary transformation by-products may be present in the starch-based biomass hydrolysates. If the hydrolysis is too short, resistant starch may occur, i.e., a form that is bonded to the fibers, which requires a longer time or more aggressive hydrolysis conditions [8].

**Figure 2:**
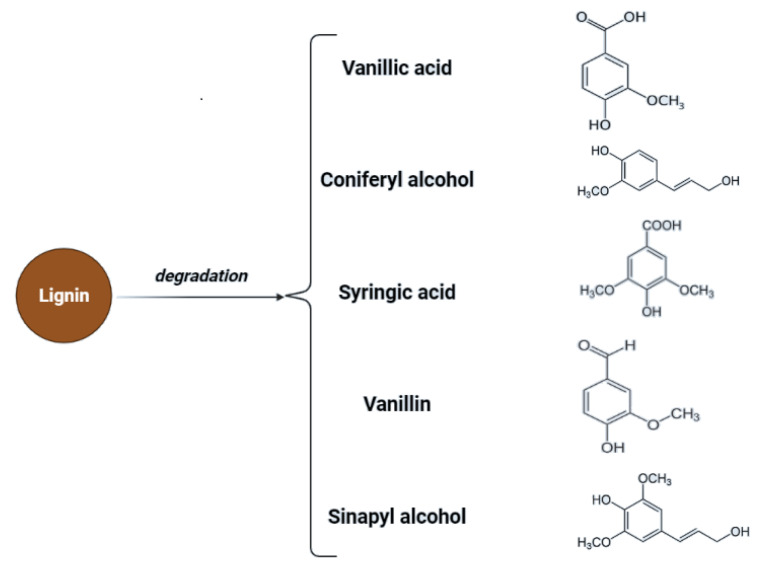
Chemical structures of monomeric compounds in the lignin structure and the main products of lignin degradation.

Cellulose is a linear polysaccharide in the form of insoluble microfibrils. Amorphous or soluble regions appear in cellulose structure, where the molecules are less compact [32]. However, cellulose fibrils are located in a lignocellulosic matrix which makes them highly resistant to enzymatic hydrolysis. The degree of polymerization (DP) changes due to pre-treatment which is associated with changes in structural parameters such as crystallinity and porosity [33]. Lou et al. showed that the DP of cellulose has a negative correlation with cellulose hydrolysis. Long cellulose chains are assumed to contain more hydrogen bonds and are more difficult to hydrolyze, while shorter cellulose chains contain a weaker hydrogen bonding system, which facilitates access to the enzyme or hydrolyzing agent [34]. Additionally, the presence and structure of hemicelluloses affect the process of saccharification, as a result of structural obstacles related to their structure. Hemicelluloses are heterogeneous groups of biopolymers [35,36] and the degree of polymerization of hemicelluloses is in the range of 100–200 units and is easily hydrolyzed by diluted acids or bases as well as enzymes [37]. Hemicellulose is considered a physical barrier restricting cellulose access. Therefore, the removal of hemicellulose could increase the enzymatic digestibility of biomass [25,38]. Additionally, lignin hinders biomass pretreatment. Lignin is a highly complex amorphous heteropolymer of phenylpropanoid monomers (p-coumaryl, coniferyl, and sinapyl alcohol) [39]. Mentioned structures are presented in Figure 2.

Lignin is responsible for hydrophobicity and structural stiffness and binds hemicelluloses to cellulose in the cell wall. The presence of lignin must be taken into account, as the secondary derivatives of lignin may affect the dark fermentation step. It is well known that lignin plays a negative role in cellulose conversion which is influenced by several factors such as total lignin content and lignin composition/structure (especially the content of hydroxyl groups). Lignin can block the access of enzymes to cellulose as a physical barrier and thus limit the access of polysaccharides [13]. Lignin permanently absorbs cellulase enzymes, therefore preventing their effect on cellulose. Adsorption of cellulases on the lignin matrix has been observed for pre-treated substrates with dilute acid or vapor explosion. It has been observed that cellulases can be adsorbed on the lignin matrix that pre-treated substrates with dilute acid or steam explosion [21].

### 2.2. Issues Related to the Availability and Construction of Biomass Surface

The accessible surface area is an important limiting factor in the cellulose digestibility process. The available surface is related to biomass particle size, porosity, and pore volume [21]. As the particle size decreases or the pore volume increases, the available surface area increases and, as a result, the enzymatic digestibility of cellulose increases. In terms of microcrystalline cellulose, it has been shown that reducing the size increases the accessible surface area and greatly accelerates the rate of cellulose hydrolysis [40]. For example, by reducing the particle size from 25.52 μm to 0.78 μm, the available surface area increases from 0.24 m^2^/g to 25.50 m^2^/g, thus increasing the hydrolysis rate [41].

Particle size is a significant parameter that affects cellulose hydrolysis potential. Some studies have shown that particle sizes smaller than 590–350 μm do not significantly improve enzymatic digestibility. However, the available surface depends not only on the particle size but also on the porosity and pore volume [42,43]. The substrate surface is divided into two external (affected by the length and width of the substrate) and internal (pore surface) which is a function of the lumen size and the number of pores and cracks under the substrate. Published studies have shown that there is a direct relationship between the inner surface and the rate of enzymatic hydrolysis and also the most important factor limiting the enzymatic digestibility of biomass is the surface area [44,45,46].

According to some studies, enzyme access to cellulose is more through the cell wall pores than the outer surface of the substrate. On average, more than 90% of the enzymatic digestibility of the substrate is conducted by available pores and the outer surface plays a lesser role [47,48]. SSA (specific surface area) is the total surface area per unit (volume or mass), and ASA (accessible surface area) represents the area at which cellulases can come into contact with cellulose. In general, ASA is directly related to SSA, and as ASA increases, so does SSA, but the whole surface is not effective for cellulose-associated cellulases, and only pores large enough can allow cellulases to take action [49,50].

Studies have shown that the enzymatic digestibility of biomass decreases after drying due to hornification and reduced pore size, so pore size can be a limiting factor in the enzymatic hydrolysis pretreatment process [51]. Hornification depends on the physical and chemical structure of the cell wall of the undried material, the drying method, and the drying time. Some studies have shown that drying significantly reduces the number of large pores and that the collapse and closure of large pores result in smaller pores that are not accessible to enzymes [52,53]. On the other hand, wet pressing to reduce the moisture content of the material causes an irreversible reduction in the volume of fiber pores and thus reduces the enzymatic digestibility of cellulose. Therefore, the effects of hornification are one of the effective factors in biomass resistance. Although SSA has an important effect on cellulose enzymatic digestibility, some factors such as cellulose crystallinity and degree of polymerase affect enzyme digestibility. As a result, these cellulose-related structures can limit the rate and extent of hydrolysis [54].

#### 2.2.1. Overcoming Cellulose Crystallinity

Cellulose has crystalline and amorphous regions and in these regions of cellulose there is a form of microfibrils in which paracrystalline groups are composed of several dozen (1, 4) β-D-glucan that are longitudinally hydrogen bonded together [7]. Due to this feature (hydrogen bonding), crystalline regions of cellulose are more resistant to enzymatic hydrolysis and microbial attacks than amorphous regions [40,47,48]. Some studies have shown that crystallinity has a negative effect on the enzymatic digestibility of cellulose, especially the initial hydrolysis rate but, on the other hand, the reconstruction of the crystalline hydrogen bonding network can increase the rate of polymerization [53]. Some researchers have shown that the conversion of crystalline allomorph Iβ to IIII by ammonia reduces the number of in-sheet hydrogen bonds of cellulose while increasing the number of inter-sheet hydrogen bonds up to five times [10].

Crystallization is considered one of the most effective and important inhibitory factors of enzymatic hydrolysis. Because the higher the cellulose crystallinity, the lower the availability of biomass for enzymatic hydrolysis. Cellulose crystallinity is caused by intermolecular and intramolecular hydrogen bonds between cellulose chains, which can be modified by biomass pretreatment methods [55,56].

Cellulose consists of two regions, amorphous and crystalline. In order to determine the crystallinity of cellulose in plants, it is very necessary to determine the cellulose content because it is expected that cellulose is the only crystalline compound [57]. Cellulose consists of linear chains of poly [b-1,4-D-anhydroglucopyranose] (C6nH10n + 2O5n + 1 (*n* = degree of polymerization of glucose)) which crystallizes through hydrogen bonding between the chains and has cellobiose as repeating units. The crystal structure of cellulose in higher plants is that of cellulose Iβ, which consists of monoclinic, P21 space groups with cellulose chains oriented along a unique c axis [58,59].

Corn and wheat straws are useful for the production of biofuel after pretreatment and enzymatic hydrolysis of cellulose and hemicellulose to monosaccharides due to their low cellulose content and large cell lumen, which causes low tensile strength. In the enzymatic hydrolysis process, exoglucanase is used for crystalline cellulose and endoglucanase is used for amorphous cellulose to convert cellulose into glucose substrate [60,61].

The effect of crystallinity on hydrolysis is different. Some studies on pretreated wheat straw [56,62], corn [63], switchgrass, and bagasse [64] reported that crystallinity is the most effective inhibitor of enzymatic hydrolysis, so the higher the cellulose crystallinity, the lower the availability of biomass for enzymatic hydrolysis. However, some studies [65,66,67,68,69,70] showed that crystallinity in limiting hydrolysis is less important than other physical properties such as the DP, pore volume, accessible surface area, and particle size.

Due to the presence of different hydrogen-bonding networks, amorphous celluloses are hydrolyzed three to thirty times faster than high crystalline celluloses [47,48,53]. In the enzymatic hydrolysis process, first, amorphous cellulose is hydrolyzed and then hydrolysis of more solid crystalline compounds takes place. However, in most studies, pure cellulose substrates have been used to investigate the relationship between crystallinity and hydrolysis rate, which does not indicate the heterogeneous lignocellulosic substrate that we encounter during the hydrolysis of pretreated substrates for biotransformation [25,26,27,71].

Physical pretreatment methods such as ball milling were used to prepare samples with different initial crystallinity degrees to show the effect of crystallinity on hydrolysis [72]. It was found a reduced particle size and an increase in the accessible surface area, which is the most important factor for the enzymatic digestibility of biomass. The most common method for determining the crystallinity of cellulose is X-ray diffraction. The crystallinity index (CrI, Equation (1)) is commonly used to describe the crystalline degree of biomass and pulp, which is defined as follows [72]:

(1) $$\mathrm{Crystallinity}\;\mathrm{index}\;\left(\mathrm{CRI}\right)\%=\frac{I_{002}-I_{\mathrm{am}}\;}{I_{002}}\times 100$$

I_002_ is the diffraction intensity of 002 peaks at 2θ ≈ 22.5° and I_am_ is the scattering intensity of the amorphous region at 2θ ≈ 18.7°.

CrI measures the relative fraction of crystalline cellulose in total solids and is affected by the presence of lignin and hemicellulose. Removal of lignin and hemicellulose increases the CrI in the pretreated material. Therefore, care should be taken when using CrI to study the effect of pretreatment processes on the change in crystallinity of biomass cellulose. Drying the sample before analysis is one of the most important limitations of using this method because drying in the air or an oven changes the crystallinity of cellulose [21].

Another method of crystallinity analysis of cellulose is the use of the infrared spectrum. Since the presence of lignin and hemicellulose can interfere with the ratio of amorphous to crystalline cellulose bonds and the ratio of crystalline cellulose polymorphs, the infrared spectrum is used for qualitative rather than quantitative studies [73].

#### 2.2.2. Degree of Polymerization

The number of glucose units in a polymer is called the degree of polymerization (DP) of cellulose. The DP plays an important role in lignocellulose resistance. By changing the DP, other structural parameters, including crystallinity and porosity, also change [9,33]. Some studies have shown that decreasing the DP in cotton by γ-radiation causes very small changes in the rate of saccharification. Long cellulose chains are assumed to consist of more hydrogen bonds that are difficult to hydrolyze, while short cellulose chains are composed of weaker hydrogen bonds that facilitate enzymatic access [34].

The process of enzymatic hydrolysis of cellulose by the synergy of cellulase components is called the process of cellulose depolymerization [15]. The endocellulase breaks down linear cellulose molecules and produces reducing and oxidizing ends that can be attacked by exocellulases or cellobiohydrolase [74]. Exocellulases then remove one of the cellulose molecular strands to create more internal sites for endocellulase binding. Cellobiose is a very strong inhibitor of the activity of endocellulase and exocellulases enzymes, and the conversion of cellobiose to glucose by β-glucosidase reduces its effect and creates the ground for continued cellulolytic activity [75].

Gupta et al. showed that endoglucanase (Endo-G) reacts rapidly with non-crystalline cellulose and reduces the DP by 30 to 60, and then Endo-G is inhibited by non-crystalline cellulose. β-Glucosidase (β-G) can hydrolyze cell-oligosaccharides with a DP less than seven and produce cellulose, while it cannot hydrolyze cell-oligosaccharides with a DP higher than seven. Therefore, due to this mechanism, the hydrolysis rate is faster in shorter cellulose chains [76].

Nahzad et al. showed that beating the pulp speeds up the hydrolysis and also showed that the initial DP of the pulp does not have a significant effect on the final amount of hydrolysis [77]. However, two-thirds of the DP decreased during hydrolysis, which was the same in all hydrolyzed pulp. The DP is similar to cellulose crystallization and is not an independent factor, because a change in the DP is always associated with a change in crystallinity. After beating, the fiber pulp becomes shorter and swells significantly with increasing porosity. As a result, biomass resistance does not arise from a single structural factor. Since the plant cell wall is made of cross-links of chemical compounds and forms a strong and compact spatial structure, there are natural interactions between these factors [77].

The overcoming of biomass recalcitrance more often involves the application of microbial consortia. Enzymes are recognized as expensive agents; their isolation and purification are expensive and complicated. Thus, they are mainly used as mixtures. Therefore, the application of wood-decomposing fungi in consortium with dark fermentation bacteria is common. Rot fungi, i.e., *Phanerochaete chrysosporium, Phlebia radiate, Dichmitus squalene, Rigidosporus lignosus,* and *Jungua separabilima* can produce lignin peroxidase, polyphenol oxidase, and magnesium-dependent peroxidase and cause the hydrolysis of lignocellulose and depolymerization [78].

White rot fungi produce three enzyme fractions [13], i.e., cellulolytic enzymes and hemicellulases (Endo-1,4-β-glucanase, Exo-1,4-β-gluconase, and glucohydrolases, endo-1,4-β-xylanases, β-xylosidases, galactoglucomannazes, or galactosides), hemicellulases; lignosaccharidases (glucose oxidase, pyranose oxidase, oxidoreductase, and cellobiase) and lignin-degrading enzymes (peroxidase, dioxygenases, peroxydismutases, and glyoxal oxidases). Soft rot fungi, i.e., *Trichoderma reesei, Chaetomim sp.* and *Ceratocystis sp., Ascomycota,* and *Deuteromycota*, are effective toward wood with high moisture content since a secondary erosion of cell walls followed by hemicellulose-cellulose complex decomposition occurs and monosaccharides are generated and consumed. The culture of this type of rot should be carried in the presence of fermentative microbes, to avoid losses of monosugars due to rot self-consumption [13].

Future directions and development regarding overcoming biomass recalcitrance by biological methods, should, to our best knowledge, consider the elimination of multistep procedures and therefore the research on the synergistic effect of rot fungi and fermentative bacteria and archaeon should be investigated.

### 2.3. Inhibitory Compounds Generation during Pretreatment

Pretreatment methods have been studied but pretreatment advances are still required since the studies were carried out mainly regarding the mono type of biomass, not biomass mixtures, characterized as more complex matrixes. Technologies of pretreatment of the lignocellulosic biomass are usually classified into physical, physicochemical, chemical, and biological processes. Pretreatment is carried out mainly due to enhancing the fermentation or biorefining processes [79].

Proper selection of a microorganism or a mixture of microorganisms and the control of the process conditions (by affecting the pH during fermentation, temperature, or oxygen content) allows the fermentation to be directed to obtain biocomponents, which are difficult to obtain in the chemical synthesis [15,42,75]. This approach creates a chance for better usage of the raw material and highlights the necessity for carrying out biorafination procedures regarding the fermentation broth [78,79]. Although biogas formed during biological processes contains hydrogen, due to different gaseous ingredients, a gaseous stream purification must be concerned. Additionally, pre- and post-fermentation broths whose composition is based on biomass hydrolysates require purification. Therefore, consideration of inhibitory by-products must be carried out [80].

Starch-based biomass processing generates a very low possibility of inhibitory compound generation. In this case, only secondary transformations of hexoses (glucose) may cause HMF generation, especially in acidic conditions [81]. Additionally, poorly chosen process conditions may lead to incomplete liquefaction of starch [8,74].

Lignocellulosic biomass has a very high potential for the production of biofuels as well as chemicals. In the case of lignocellulose and saccharification, the conversion of complex carbohydrates molecules into simpler sugars is required [79]. Inhibitors may be formed during the hydrolysis process—mainly lignin derivatives—and as secondary transformation products due to saccharification products’ transformation under specific conditions. To maximize the fermentation of hexoses (C_6_) and pentoses (C_5_), and to minimize the presence of inhibitors during the fermentation process, the concentration of possible derivatives must be monitored throughout hydrolysis [82]. Transformation products of pentoses and hexoses include furfural and hydroxymethyl furfural (HMF), considered fermentation inhibitory compounds. For possible lignin derivatives, please refer to Figure 2.

The type of chemical used in the pre-treatment process can have different effects on the structural components of lignocellulose. For example, alkaline pretreatment, ozonolysis, peroxide, and wet oxidation are more effective at removing lignin, while dilute acid pretreatment is more effective at removing hemicellulose [80,81,82].

#### 2.3.1. Inhibitors Generated during Acidic Pretreatment

Mineral acids such as H_2_SO_4_ can be used to pre-treat lignocellulosic biomass. Depending on the dose of acid used in the process, it can be divided into concentrated or dilute acid hydrolysis. In the concentrated acid hydrolysis method, lignocellulosic biomass with high concentrations of sulfuric acid is purified at ambient temperature, which results in high sugar yields. The use of this method has the advantage of not using enzymes for saccharification. However, this process also has disadvantages, such as corrosion of equipment, high acid consumption, long reaction time, as well as acid recovery after purification, which to some extent causes limitations in using this method. In the second method, using dilute acid (0.5–1% H_2_SO_4_) and high temperature can convert cellulose to glucose [83].

The high-temperature conversion of cellulose to glucose is an efficient way to achieve an acceptable rate of hexoses. This method, despite the low concentration of acid, short reaction time, and application of high temperature, accelerates the decomposition of hemicellulose sugars while increasing the corrosion of equipment due to the formation of inhibitory by-products and the need to neutralize the pH for subsequent processes problem [15]. Pre-treatment using the dilute acid method can hydrolyze 100% of the hemicellulose to its constituent sugars depending on the pre-treatment conditions. The main purpose of pretreatment with dilute acid is to increase the sensitivity of cellulose to microbial degradation and enzymatic hydrolysis [84]. A two-step process can be used to prevent the decomposition of sugars. In the first stage, hemicellulose sugars are released under mild conditions, and in the second stage, cellulose-rich solid residues are released under more severe conditions. Depending on the nature of the lignocellulosic material, temperatures of 140 to 190 °C are used for the first stage and 190 to 230 °C for the second stage [84,85].

**Figure 3:**
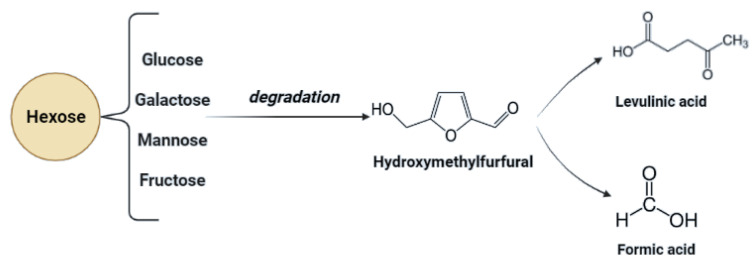
Chemical structures of inhibitory compounds are generated due to secondary transformations of hexoses during acidic pre-treatment.

In the process of acid hydrolysis, temperatures above 110 °C cause the formation of toxic inhibitory compounds such as furfural and 5-hydroxymethyl furfural [79]. These compounds inhibit enzymatic and microbial hydrolysis. Their removal is necessary and possible adsorption on activated carbon or precipitation with calcium hydroxide. Other inhibitors such as chloric, phosphoric, or nitrous acids can be formed with increasing temperature and depend on the hydrolyzing agent and pollution of biomass with inorganic pollutants [13,86]. Acidic pretreatment generates inhibitors that must be removed to minimize downstream processing costs. In Figure 3, possible inhibitory compounds occurring due to cellulose monomers degradation are presented.

#### 2.3.2. Inhibitors Generated during Alkaline Pretreatment

Alkaline pretreatment of lignocellulosic biomass is one of the most effective methods to increase the concentration of reducing sugars in the hydrolysis process [29]. In alkaline pretreatment, dilute bases such as sodium, potassium, calcium, hydroxides, and ammonia are used in the treatment of lignocellulosic biomass, of which sodium hydroxide is the most common alkali. Alkaline processes use less temperature and pressure than other methods [87,88].

**Figure 4:**
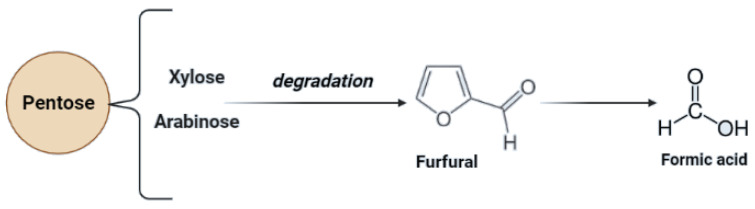
Chemical structures of inhibitory compounds are generated due to secondary transformations of pentoses during alkaline pre-treatment.

This process improves the digestibility of cellulose but the decomposition of sugars in this method is less than in acidic pretreatment. However, the main obstacle to this method is the high cost of alkalis. The application of calcium hydroxide, due to its low cost and ability to recover or regenerate ammonia, which is recyclable due to volatility, can be used as a solution to this problem [83,89]. Alkaline pre-treatment also enables hemicellulose degradation. In this case, pentoses may occur [79]. Due to secondary transformations during the hemicellulose structure degradation, secondary derivatives of pentoses may occur (Figure 4).

#### 2.3.3. Inhibitors Generated during Oxidative Pretreatment

Hydrogen Peroxide

In the oxidation pretreatment process, peroxides such as hydrogen peroxide or alcoholic solutions of acetic acid are used. Oxidizing agents can dissolve amorphous cellulose and lignin, while hemicellulose can be dissolved when separated from the biopolymer. Crystalline cellulose is not dissolved in this method. In this method, processes such as electrophilic substitution, site chain dislocation, and aryl-alkyl bonding cleavage occur [90].

Alkaline hydrogen peroxide is used in the paper industry as bleach, lignin, and xylene remover. This process is very mild and leaves no contamination in the lignocellulosic biomass and also decomposes into water and carbon dioxide [13,79]. In addition, in this process, no by-products and inhibitors of pentose and hexose decomposition are formed. The oxidizing pretreatment reduces the biomass resistance. The hydrogen peroxide only reacts with the aliphatic biopolymer compounds, and in alkaline conditions, due to the presence of the cumene anion, lignin is separated from the lignocellulose structure [91]. Unfortunately, the reagent is unstable under alkaline conditions and decomposes easily in the presence of transition metals such as Mn, Fe, and Cu, so the application of hydrogen peroxide requires special processing conditions, which is hard to accomplish in bio-fraction mixtures. The highest pH value-enabling efficient alkaline pretreatment with H_2_O_2_ is 11.5. The lowest applicable hydrogen peroxide concentration is 1% with a mass proportion between H_2_O_2_ and biomass equal to 1: 4 [92].

Wet Oxidation Process

In the wet oxidation process (WO), water and oxygen or air with high pressure and temperature above 120 °C are used for the pre-treatment of lignocellulosic biomass. Combining the alkaline process with wet oxidation, in addition to accelerating the oxidation rate of lignin, prevents the formation of furfural and inhibitory compounds [83]. The WO can be used as an effective pretreatment method to convert lignocellulosic biomass, such as wheat straw, to a soluble hemicellulose fraction and a solid part with high cellulose content with high sensitivity to enzymatic hydrolysis. In this process, the acids produced due to the dissolution of hemicellulose components catalyze subsequent hydrolytic reactions, which decompose hemicellulose into components with low molecular weight and that are soluble in water. At high temperatures, lignin degradation is particularly important because phenolic compounds and carbon–carbon bonds are highly reactive under wet oxidation conditions. In this process, lignin is broken down into CO_2_, H_2_O, and carboxylic acids, which may be the sole carbon source for photo fermentative bacteria [93].

Ozonolysis

In this method, ozone is used to dissolve lignin and part of hemicellulose. The process of ozonolysis at room temperature can remove lignin without producing any toxic or inhibitory compounds. The main limitation of using this method is the cost [83].

### 2.4. Summary of Pre-Treatment Methods Advantages and Disadvantages

In Table 2, the advantages and disadvantages of mentioned pre-treatment methods are summarized [13,74,78,86,94,95,96,97].

The comparison of the advantages and disadvantages of commonly used pretreatment methods indicates that there is no universal method that will create effective pretreatment options for each known type of biomass. The selection of the appropriate method must be empirical and should take into account the issues related to the processing of the raw material, but also consider the by-products of this treatment. Making it necessary to detoxify the liquefied parts of biomass will significantly affect the technological effectiveness of fermentation processes and their efficiency. In addition, after the fermentation processes, it may be necessary to clean the gas streams from inorganic impurities related to the pre-treatment method used, but also from liquid streams in difficult-to-remove derivatives of hydrolyzed polymers and their decomposition products.

## 3. Bioconversion of Hydrolysates

### 3.1. Hydrogen Generation

In nature, anaerobic microorganisms produce hydrogen gas in the absence of oxygen and use the phenomenon of fermentation, but the amount of this gas is low and is not economically justifiable for industrial and domestic use. Therefore, it is necessary to search for methods to increase the efficiency of hydrogen gas production [15]. Anaerobic bacteria generate biohydrogen and organic acids via dark fermentation. The biohydrogen from dark fermentation requires purification and the organic acids may be applied as a sole carbon source in photo fermentation. *Rhodospirillum rubrum* is a frequently studied species, which exhibits unique nitrogenase activity, reducing both molecular nitrogen and protons to molecular hydrogen via photo fermentation [17]. The microorganisms that synthesize biohydrogen in the photo fermentation process with the use of hydrogenase also include purple sulfur bacteria, which are strict anaerobes, e.g., *Allochromatium vinosum, Thiocapsa roseopersicina, Chlorobium vibloroforme, Desulfuromonas acetoxidans,* and *Chloroflexus aurantiacus*. The range of electromagnetic radiation waves absorbed by them ranges from 400 to 950 nm. *Rhodospirillum rubrum*, the commonly used microorganism in photo fermentation is classified as mesophilic, and its temperature optimum is 25–30 °C. It has polar flagella and is a facultative anaerobe [13,17]. Depending on the presence of oxygen, it can carry dark fermentation or oxygen respiration. It is also capable of photosynthesis as it contains carotenoids and bacteriochlorophyll. In addition to the ability to bind carbon dioxide, it can bind nitrogen. It contains both Fe-Mo- (iron-molybdenum) and Fe-nitrogenase. Microorganisms of this type are currently one of the most promising in the field of biohydrogen synthesis by photo fermentation [18].

The main purpose of starch and lignocellulosic pretreatment processes is to reduce the degradation of sugars, minimize the formation of inhibitory compounds, and reduce the consumption of chemical compounds, energy, water, and waste production [98]. Additionally, the digestibility in bioconversion of biomass must be improved as an effect of biomass pre-treatment

The efficiency of bioconversion concerning hydrogen generation for diversified sole carbon sources in the broths is presented in Table 3.

As seen in Table 3, most research carries the discussion on fermentation efficiency on a single substrate. A review of the literature has shown that there is a lack of research based on the fermentation of real mixtures and real wort, in which lignocellulosic and starch polymers can occur simultaneously as carbon sources. Industrial practice in the field of hydrogen fermentation is that in the case of biohydrogen, there must be a departure from the fermentation of only one raw material towards the co-fermentation of many raw materials. This type of approach is more and more often ordered and fits into the assumptions of the circular economy.

**Table 3:** The efficiency of bioconversion of biomass hydrolysates concerning applied microorganisms and the main sole carbon source present in the fermentation broth.

Substrate	Amount	Organism	Reactor type	pH	Temperature (°C)	HRT	Hydrogen Productivity	Hydrogen Yield	COD Removal (%)	% H_2_	Reference
Glucose	10 g/L	Clostridiaceae Flexibacteraceae *	Membrane Continuous	5.5	35	3.3	640 mL H_2_/(L·h)	4 mol H_2_/mol glucose	-	60	[99]
Sucrose	10 g/L	E. cloacae IIT-BT 08 *	Batch	6	36	-	660 mL H_2_/(L·h)	6 mol H_2_/mol sucrose	-	92	[100]
Glucose	1%	E. cloacae *	Batch	6	36	-	447 mL H_2_/(L·h)	2.2 mol H_2_/mol glucose	-	-	[100]
D-Xylose	10 g/L	E. cloacae IIT-BT 08 *	Batch	6	36	-	348 mL H_2_/(L·h)	0.95 mol H_2_/mol xylose	-	-	[100]
L-Arabinose	10 g/L	E. cloacae IIT-BT 08 *	Batch	6	36	-	360 mL H_2_/(L·h)	1.5 mol H_2_/mol arabinose	-	-	[100]
Glucose	10 g/L	Mixed culture from compost	Batch	5.5	60	-	147 mL H_2_/(L·h)	2.1 mol H_2_/mol glucose	-	-	[101]
Glucose	20 g COD/L	*Clostridia* sp. *	CSTR Continuous	6	28–32	6	7.42 mmol H_2_/(gVSS·h)	1.42 mol H_2_/mol glucose	-	43	[102]
Glucose	7 g/L	Mixed culture	CSTR Continuous	5.5	36	6	-	2.1 molH_2_/mol glucose	-	64	[103]
Glucose	4.85 g COD/L	Mixed culture	UASB Continuous	7.2	70	26.7	11.15 mmol H_2_/d	2.46 mol H_2_/mol hexose	-	55	[104]
Sucrose	20 g COD/L	Mixed culture	Immobilized bed Continuous	6.7	35	1	1.32 L H_2_/(L·h)	-	-	34	[105]
Sucrose	1 g COD/L	Mixed culture	Batch	6	26	-	-	1.8 mol H_2_/mol sucrose	-	-	[106]
Sucrose	20 g COD/L	Mixed culture	CSTR Continuous	6.7	35	8	0.105 mol H_2_/h	3.47mol H_2_/mol sucrose	-	42	[107]
Sucrose	25 g/L	Mixed culture	Fermenter Batch	5.5	35	-	1504 mL H_2_/h	2 mol H_2_/mol glucose	-	-	[108]
Lactose	29 mmol/L	C.termolacticum *	CSTR Continuous	7	58	35.7	2.58 mmol H_2_/(L·h)	1.5 mol H_2_/mol hexose	-	55	[109]
Glucose	5.5 g/L	Enterobacter aerogenes *	Bioreactors		42	48	25.44 mL/g biomass 283.45 _ 1.87 mL/YTRS	-	-	-	[26]
Paulownia	-	photosynthetic consortium HAU-M1 *	-	7	30	26–38	338.41 mL	67.11 mL/g	62	-	[110]
Xylose	20 g/L	*Lactobacillus* and *Sporolactobacillus* spp., *Clostridium* sp *	Dynamic membrane module bioreactor (DMBR)	7.5	37	3–12	30.26 L H_2_/L-d	1.40 mol H_2_/mol xylose	-	-	[111]
Glucose	10 g/L	Caldicellulosiruptor *	Batch	-	70	20	10.55 mmol/L/h	4 mol H_2_/mol glucose	-	-	[112]
Glucose and Xylose	50 % mol/mol	Rhodopseudomonas palustris *	Bioreactor	7	30	-	30.6 mL h^−1^ L^−1^	1.63 (mol H_2_/mol carbon)	-	-	[113]
Glucose, xylose	41.17 g/L	Rhodospirillum rubrum *	Batch	4.5	60	-	819 mL H_2_/L medium m/7 d	-	82	-	[114]
Rice husk	5 g dw	Clostridium termitidis ATCC-21846 * and *Clostridium intestinale ATCC-BAA* *1027*	Batch	7.5	37	-	0.023 mL H_2_ g^−1^ dw rice husk h	5.9 mL g^−1^ dw Rice husk	-	29.26 mL	[115]
Corncob	10 g	HAU-M1 * photosynthetic bacteria	Batch	7	50	48	-	27.34 mL/g TS	-	80.94	[116]
Duckweed and corn straw	5:1	photosynthetic strain *HAU-M1*	Batch	8	30	18.57	-	85.6 mL/g TS	-		[117]

* The kind of bacteria.

Co-fermentation uses a mixture of biowaste from different sources. Joint fermentation allows to obtain proper hydration of the fermentation mass, improvement of the balance of biogenic elements, or an increase in the load of easily biodegradable matter, which contributes to a more stable course of the process, and also allows for the synergy effect, which increases the efficiency of organic mass decomposition and biohydrogen generation efficiency.

The most frequently tested dark fermentation feeds are hydrolysates based on glucose and sucrose. A review of the literature [26,99,100,101,102,103,104,105,106,107,108,109,110,111,112,113,114,115,116,117] showed that for most model studies, substrate concentrations of about 10 g/L were neutral towards the slightly acidic pH of the fermentation broths. Only a few studies [104] were carried out for a pH > 7; however, as it appears from the content of the study, this is only the starting pH, which drops during fermentation due to the formation of organic acids during fermentation in a continuous UASB reactor. Researchers of processes mentioned in Table 3 have not decided to conduct continuous processes, but there are exiles with two-stage fermentation or in a continuous rotary system. Based on experiments, it can be concluded that in most cases when pure cultures [26,100,111] are used, slightly higher yields of hydrogen can be obtained than when mixed cultures are used [104,105,106,107,108]. Unfortunately, the nature of these pure cultures often requires the use of relatively high temperatures, since they are thermophilic strains. Based on the published data, it is impossible to balance the productivity of hydrogen and the energy sense of the processes performed. Therefore, tests for real broth conditions are required, to compare the effectiveness of the fermentation data or to adjust a universal model for fermentation conditions. This kind of model must raise issues related not only to gaseous products but also to the composition of the post-fermentative liquid. When increasing the profitability of the process is possible, more attention is paid not only to detoxification but also to potential methods of post-fermentation broth management.

### 3.2. Genetic Modifications of Microorganisms

Genetic modification of microorganisms is an interesting aspect of enhancing hydrogen productivity with the simultaneous adaptation of microorganisms against the resulting fermentation by-products. Industrial conversion of biomass to fuel currently involves heat and chemical treatment to overcome the biomass recalcitrance of starch, cellulose, hemicellulose, and lignin, followed by enzymatic hydrolysis to dissolve the plant cell wall to produce a fermentable substrate for fuel-producing microorganisms. All of these methods add costs and, on the other hand, produce hydrolysates that are toxic to microorganisms and harmful to the sugars in biomass [118]. An approach involving genetic engineering techniques to manipulate the metabolism of microorganisms may also be applied. The efficiency of biohydrogen production can be improved and, consequently, the total processing costs can be reduced. Expression of the genes responsible for the production of organic acids may be turned off, while the strains with multiple enzyme systems, including cellulase and xylanase, which are responsible for the breakdown of cellulose and hemicellulose, may be introduced and developed [15]. Genetic engineering provides the basis for increasing biohydrogen production. There are several methods, including deletion of a competitive gene, overexpression of a homologous or heterologous gene, creation of artificial pathways, culture, and identification of indirect hydrogen-producing organisms to improve hydrogen metabolic function through genetic engineering. A genetic modification of enzyme activity can be effective when the specific amount of that enzyme is limited [119]. The possible paths and the effects of genetic engineering of different species of microorganisms on biohydrogen production efficiency are presented in Table 4.

To the best knowledge of the authors, research in the field of genetic modification of microorganisms for adaptation to the composition of hydrolysates is not currently carried out. This type of approach could, however, allow the implementation of large-scale processes and independence from difficult ingredients in fermentation broths, which may occur as a result of pre-treatment. However, the designed genetically modified microorganisms have to be tested on real fermentation broths since research has shown that the adaptation of model conditions to real broths may require time-consuming optimization.

## 4. Post-Fermentative Broth Detoxification and Management Methods

The inhibitory compounds generated during pre-treatment may affect the efficiency of bioconversion; the broth composed mainly from the hydrolysates must be processed for detoxification, if necessary [79].

The degrees of inhibition of lignocellulosic hydrolysates and also the degree of inhibition tolerance of various microorganisms are different. The choice of detoxification methods depends on the source of lignocellulosic hydrolysate and the microorganisms used. Therefore, detoxification methods can be divided into three groups: physical, physicochemical, and biological [135].

As chemical structures are a criterion for classification, inhibitory compounds may be divided into four groups [79]: substances produced by hemicelluloses (acetic acid which is the source of deacetylation of xylan); substances that are produced from the degradation of lignin (phenolic compounds and other aromatic compounds); materials obtained from the destruction of pentoses (furan derivatives, furfural) and hexoses (5-hydroxymethylfurfural); And metals leached from inorganic pollutants and/or equipment (copper, chromium, nickel, and iron) [101,114]. All of these compounds can individually or synergistically affect the physiology of fermenting microorganisms during bioconversion [134]. Therefore, the removal or reduction of the amount of these compounds is necessary to increase the efficiency during the microbial fermentation process of biomass hydrolysates [135].

**Figure 5:**
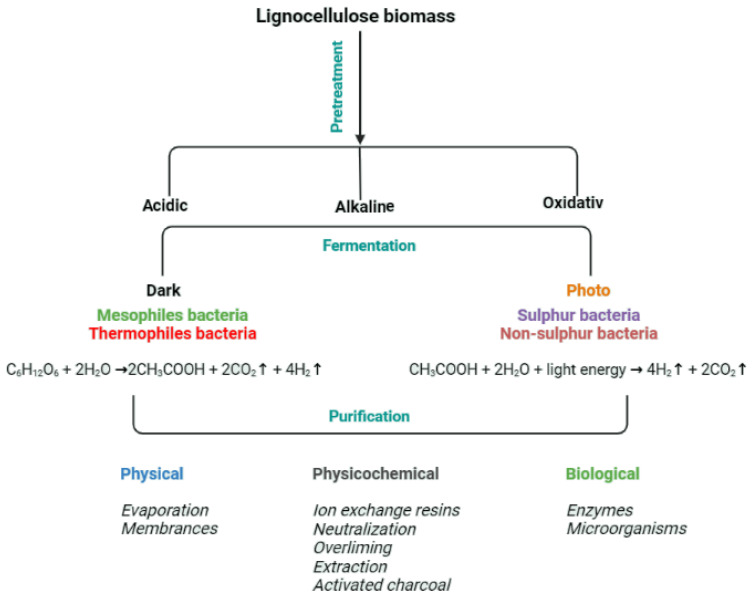
The procedure of biomass pre-treatment and the applied methods for broths purification.

The processing of post-fermentation broths may be crucial for their management, especially since the broth may contain not only the products of microbial metabolism but also remnants of the microbes present in the broth during bioconversion. The methods of purification of the remnant broths before and after fermentation would therefore be the same, and the applicable ideas are presented in Figure 5.

### 4.1. Physical Methods

#### 4.1.1. Evaporation

The evaporation process can be used to remove toxic inhibitory compounds, such as acetic acid, furfural, and vanillin. One of the disadvantages of this method is the increase of non-volatile toxic compounds as extractives [134]. Evaporation may be used especially for post-fermentation broth purification, since an increase in the temperature may affect the structures of microbes in the broth. Increased temperature causes the denaturation of proteins and, therefore, allows for the removal of the proteins and microbial remnants from the post-fermentation broth, especially when coupled with centrifugation and sediment separation.

#### 4.1.2. Membranes

The membrane process can be used as one of the detoxification methods. This method prevents the aqueous phase (hydrolyzate) from mixing with the organic phase (solvents) which is toxic to microorganisms [136]. Each membrane has surface functional groups that can remove inhibitory compounds such as acetic acid, 5-hydroxymethyl furfural, furfural, formic, levulinic, and sulfuric acids from hydrolysate solutions [137].

### 4.2. Physicochemical

#### 4.2.1. Ion Exchange Resins

The process of ion exchange resins is one of the most effective detoxification methods. In this process, inhibitory compounds derived from lignocellulose hydrolysis, including lignin, acetic acid, and furfural, are removed, thus improving the efficiency of the fermentation process [138]. The main advantage of this method is that they are recoverable and can be reused without affecting the detoxification efficiency. On the other hand, this method also has disadvantages, such as increased high-pressure drop across the bed during work, long processing time due to slow pore diffusion, and the possibility of degradation of fragile biological product molecules, In this method, a significant amount of fermentable sugars are lost after the process [115].

#### 4.2.2. Neutralization

Because the pH is low after acid hydrolysis pretreatment, the pH neutralization process approaches the fermentation conditions. Additionally, the inhibitory compounds (phenol and furfural) are removed by precipitation. This method uses chemical compounds such as calcium hydroxide and sodium hydroxide. In the calcium hydroxide neutralization method, CaSO_4_ precipitates are produced which must be removed from the environment by centrifugation in the next step, so the production of precipitates can cause problems in the fermentation process [139].

#### 4.2.3. Overliming

Among detoxification methods, the CaSO_4_ process has been reported as one of the most widely used methods [140]. In this process, first, the pH of acidic hydrolysis increases and then decreases to the desired pH for fermentation. During this pH increase, toxic, inhibitory, and unstable compounds precipitate. This method has high efficiency in removing these compounds, especially furan compounds, and is an economically desirable method [141,142].

#### 4.2.4. Activated Charcoal

Activated charcoal is another widely used method for detoxification. This method is very low-cost and efficient. In this method, most phenolic compounds are removed and also do not cause many changes in the level of fermentable sugars. Important factors in improving this process are the ratio of activated carbon to hydrolyses, pH, temperature, and contact time [135,143].

#### 4.2.5. Extraction

Solvent extraction is an efficient method for the removal of highly available toxic inhibitory compounds such as acetic acid, furfural, vanillin, hydroxy-benzoic acid, and low molecular weight phenolics. Ethyl acetate, chloroform, and trichloroethylene are among the most common solvents used in this process [144].

### 4.3. Biological Methods

In the biological process, special enzymes and microorganisms are used to remove or induce changes in the composition of the inhibitory compound [134]. The advantages of this method include the following: less waste production, the possibility of detoxification in the fermentation vessel, being environmentally friendly, fewer side reactions, and lower energy requirement [145].

This group of methods requires a long process time. Enzymes such as laccase and peroxidase derived from white-rot fungi are used to remove phenolic compounds from lignocellulose hydrolyzers. The main mechanism of detoxification of these enzymes may include oxidative polymerization of low molecular weight phenolic compounds. White rot fungi may be applied both in pre-treatment and broth management steps. They also catalyze the oxidation of alternative phenols, anilines, and aromatic thiols [146,147]. Another disadvantage of enzymatic detoxification is the long incubation time and high cost. While the advantage of this method is that it takes place in mild environmental conditions (neutral pH and mesophilic temperature) [148].

Microorganisms such as yeasts, fungi, and bacteria can be used to absorb inhibitory and toxic compounds. Some microorganisms can release cellulose and hemicellulose during incubation and only decompose lignin, so this method creates a lignocellulosic substrate that can decompose fermentable sugars in a mild and short time [146,148].

### 4.4. Perspectives of Broth Detoxification and Management Methods

Known methods of post-fermentation broth management focus primarily on lowering those parameters of the liquid, which may affect the possibility of discharging the broth to wastewater. Most often this includes metal ion content, total organic carbon level, chemical and biological oxygen demand, solids content, and phenolic compounds content. The authors, however, have some experience in model post-fermentation broth management [149] which, to our best knowledge, may be adopted under real conditions.

Interest in the topic of broth management methods has shifted to the possibility of extracting added-value products from fermentation broths [134,135,136,137,138,139]. If for some reason, on the side of microorganisms, the complete utilization of monosaccharides by fermentation is not achieved, it is possible to control the detoxification process in such a way as to convert monosaccharides into substances with potential use in the synthesis of green solvents [149]. One must remember that the post-fermentation broth may contain some inhibitors present due to pre-treatment residual products, but also other chemicals that have been recognized as fermentation inhibitors that were generated during the fermentation process. Some of these chemical compounds could become precursors of deep eutectic solvents that can be obtained in situ, directly from post-fermentation broths.

The pilot research carried out by the team [149] indicates that this type of action allows us to successfully obtain DES based on LA, HMF, and furfural. At the same time, since this process requires the conversion of monosaccharide residues, which is carried by increasing the temperature and reducing the pH, the microorganisms die and decay, and the proteins suspended in the broth are denatured. It is therefore possible to generate DES in situ and to precipitate morphotic cell debris. The treatment of the broth by precipitation of solid particles and their subsequent centrifugation allows at the same time to reduce the parameter related to the content of solid particles, reducing the values of COD and BOD. Only after the separation of the liquid and solid streams, the pre-processed fermentation broth, often rich in organic acids being a product of the metabolism of dark fermentation bacteria, can be directed to photo fermentation, where the organic acids will be processed by microorganisms, i.e., the *Rodospirillum rubrum* sp [114].

A promising direction of broth management is biorefining, which allows, under specific conditions, to obtain a wide spectrum of end products. Biorefining mainly concerns raw materials of plant origin and the process requires the separation of botanical and chemical components [18,31,46,99]. After the processing of biomass to obtain hydrogen, the remaining debris, solids, and liquids can be converted from one form to another. Such transformations require the use of enzymes, microorganisms, and other biological agents. As a result of the biorefining process, plant-specific ingredients, such as proteins and lipids, are recovered from plants [46].

The disposal of post-fermentative broth during a co-fermentation in a biohydrogen plant allows carrying sufficient management of the broth. After dark and photo fermentation, several nutrients and microelements still occur in the broth. Therefore, an addition of post-fermentation broth to a co-digester may have a promoting effect on methane generation. Finally, the leftovers from the biohydrogen plant may be applied as fertilizers after maturation.

Future perspectives should also consider problems related to scale-up, for each discusses the pre-treatment method. These types of problems arise in the very first part of the technology when pre-treatment is considered. Acidic pretreatment with the application of high acid concentrations causes corrosion of the equipment, which can involve a lot of maintenance and repair costs. Due to the low pH, it requires the neutralization of hydrolyzed biomass before fermentation. Due to the production of inhibitory compounds, it needs to add a detoxification step to remove these compounds, thus the costs will be increased. Optimization of the process parameters before scale-up is required. Alkaline pretreatment with the use of sodium, potassium, calcium, and ammonia hydroxide in high concentrations has a very high cost and, on the other hand, their entry into the environment causes environmental problems, so there is a need for recycling, wastewater treatment, and waste handling that will increase the costs. Oxidative pre-treatment is cost-consuming and requires special processing conditions and safety regulations. Biological pretreatment requires specific control for the growth conditions of microorganisms and a skilled operator. It needs a lot of space and time since it has a low efficiency compared to other methods. Enzymatic pretreatment requires optimal conditions and constant monitoring of hydrolysis temperature, time, pH, enzyme loading, and substrate concentration, so it has a difficult design and operation. During ionic liquids pretreatment, the recovery of the hydrolyzing agent and the reuse of ionic liquids are major problems for industrial applications of biomass pretreatment. As supercritical CO_2_ pretreatment is considered, the use of high temperature and pressure is an important economic problem on a large scale. DES pretreatment is very complicated due to the responsibility of many variables, so skilled operators are needed and, on the other hand, the high viscosity of DES severely limits their use.

## 5. Summary

Investigations on a multistep bioconversion process of biohydrogen generation should be carried out concerning the sustainable development and minimization of energy expenditure for the purification of post-production streams. Most published papers focus on the purification of gaseous streams after fermentation, since the generation of biofuels is the main direction of the conducted studies. Since several intermediate liquid streams occur during the multistep process of bioconversion, their disposal, detoxification, and management must be taken into account. Hydrolysates of starch and lignocellulose may become a source of vanillin, syringole, cumarol, furfural, HMF, levulinic acid, and many other value-added products. The literature lacks models that would allow us to unambiguously balance the productivity of biohydrogen, and at the same time assess the environmental effect of the fermentation carried out with the potential liquid pollutants generated as a result of the conducted fermentation.

Several paths of pre-treatment and derivatives occurring in the broth due to this process were discussed. According to the literature, the purification of intermediate streams does not differ from the purification after the bioconversion and, therefore, if the applied temperature does not increase above the temperature allowing for protein denaturation, it may be carried to obtain liquid streams allowing to improve the bioconversion to gaseous biofuels, i.e., biohydrogen. On the other hand, the post-fermentation broth after dark fermentation contains organic acids and therefore may be purified by subjecting it to photo fermentation. This approach allows us to improve the overall efficiency of hydrogen. The insights of the process based on broths composed of organic acids should become a significant direction of research.

In the opinion of the authors, the used biomass pre-treatment method influences the possible paths of managing the fermentation broth. If the possibility of using diversified raw materials, different cultures, and process parameters is considered, it turns out that despite the multitude of research in the field of fermentation, there is no universal method that would allow the management of all types of broth. This justifies the need for further research, an important aspect of which is a complete approach to the topic, which will enable the description of each of the streams and an unequivocal way of its management. Many investigations are focused on finding a novel bio-refining path that would allow for improving the economic issues in biofuel generation. Methods allowing the generation of green solvents, i.e., DES, or allowing the disposal of post-fermentation broth as an addition in a biohydrogen plant and as a fertilizer precursor seem promising.

## Figures and Tables

**Table 1:** Main features affecting the course of pretreatment of starch and lignocellulose raw materials.

Criterion	Starch	Lignocellulose
Microbial resistance	Biodegradable, excluding granular amylase-resistant α-glycans	Resistant to biodegradation due to the strong and compact structure of plant cell walls
Factors affecting access to monosugars	Fiber, physical form	Available surface, pore size, volume, particle size, specific surface area, and degree of polymerization
Chemical compounds or units	Glucose monomers linked with 1,4 and 1,6 linkages; linear polymer—amylose; branched form—amylopectin.	Lignin (amorphous heteropolymer of phenylpropanoid building units, i.e., p-coumaryl, coniferyl, and sinapyl alcohol); hemicellulose (various monosaccharide subunits to form xylans, xyloglucan, mannans, and glucomannans); cellulose (ß-D-glucopyranose units linked via ß-(1,4) glycosidic bonds, with cellobiose as the fundamental repeating unit), extractives
Chemical interactions between polymers	α -1,4 glycosidic bonds;α-1,6 glycosidic bonds	Hydrogen bonds (between cellulose-hemicellulose); Lignin-carbohydrate complex, i.e., the occurrence of phenyl glycosides, γ-esters, benzyl ethers, ferulate esters, coumarate esters; Hemiacetal and acetal linkages at 4-OH and 4-O positions (lignin covalently linked to hemicellulose)
Possible pre-treatment by-products	Monosaccharides conversion by-products and secondary transformation products, i.e., levulinic acid, HMF, furfural	Lignin derivatives, HMF, vanillin, syringole, furfural, p-coumaryl, coniferyl, sinapyl alcohol, oligopeptides, terpenoids, and levulinic acid, monosaccharides conversion by-products, i.e., levulinic acid, HMF, furfural

**Table 2:** Summary of chosen advantages and disadvantages of biomass pre-treatment methods.

Pre-Treatment Method	Advantages	Disadvantages
Enzymatic hydrolysis	The precise method for saccharification, the possibility of process planning, and the selection of an enzymatic liqueur for a specific raw material	The process parameters must be carefully designed and controlled. Enzymes are expensive and not always able to recirculate, loss of activity if the local temperature is unstable, and some saccharification by-products are recognized as enzyme inhibitors
Biological pre-treatment	Ability to design a microbial consortium, reducing the number of pre-treatment steps. Allows the design of a precise liqueur of enzymes at lower costs, a wide range of process parameters, and the possibility to obtain wild rot species for precise raw material	Time-consuming process, the possibility of monosaccharide self-consumption if the consortium is designed inappropriately, risk of infection of the bacterial culture, difficulties in separating the products, and toxicity of the fermentation broth; work in a two-phase system is necessary
Acidic hydrolysis	A method that is cheap, easy to control, widely used, and allows comparison of the results of the pre-treatment. The application of a wide range of acid concentrations allows for controlling the generation of inhibitory compounds	High temperatures, specific requirements of reactor materials, decomposition of parts of the main product and its transformation into inhibitors, and emission of oxides as a result of the fusion of acid particles
Alkaline hydrolysis	An effective method to increase the concentration of reducing sugars in the hydrolysis process. Alkaline processes use less temperature and pressure than other methods	The decomposition of sugars in this method is less than in acidic pretreatment. Possibility of fermentation inhibitors generation and secondary transformation of saccharification products
Oxidative hydrolysis	Dissolves amorphous cellulose and lignin, possibility to remove lignin without derivatives generation, COD lowering effect	Reagents are unstable under alkaline conditions and decompose easily in the presence of transition metals and the application of hydrogen peroxide requires special processing conditions
Ionic liquids	It is an effective method for dissolving the plant cell wall that does not require high temperature to dissolve the cell wall. This method is used in mild processing conditions. It also has low volatility and reusability, selective removal of lignin and hemicellulose as well as cellulose release	Failure to recycle solvents creates toxic substances in the environment and deactivates enzymes
Supercritical fluid CO_2_Water	The decomposition of sugars is low and, unlike acid methods, the amount of corrosiveness is significantly reduced. It prevents the degradation of xylose at low temperatures, recovers, and reusesNo need to dry biomass before pretreatment and reduces resistance to mass transfer. Requires a very short reaction time; therefore, the decomposition of glucose, xylose, and arabinose sugars is prevented.	Requires high pressure and temperature, non-change of lignin and hemicellulose, increasing the concentration of xylan and furan for pretreatment of corn
DES	High recovery of sugars during the pre-treatment process, improving the rate of enzymatic saccharification, preventing the degradation of polysaccharides, and preserving carbohydrates. Excellent performance on lignin extraction and biomass saccharification enhancement. Ability to selectively dissolve lignin and hemicellulose	The high viscosity limits their application and the pretreatments are often very complex, with the inhibition effect toward cellulase and acidic DESs destroying polysaccharides

**Table 4:** Effect of genetic modification in various species of bacteria on hydrogen efficiency in bioconversion.

Microorganism	Strain	Genetic Modification	mol H_2_/mol Glucose	Reference
Caldicellulosiruptor bescii *	-	deletion of L-lactate dehydrogenase gene (ldh)	2.5	[118]
Escherichia coli *	SR15	modifying ΔldhA, ΔfrdBC	1.82	[120]
Escherichia coli *	-	production BW25113 hyaB hybC hycA fdoG frdC ldhA aceE	2	[121]
Escherichia coli *	MC4100, wild-type FTD89, mutant	deletion of Hyd-1 + Hyd-2; hyaB + hybC	1.043	[122]
Escherichia coli *	FTD67, mutant	deletion of Hyd-2; hybC	1.024	[122]
Escherichia coli *	W3110, wild-type SR15, mutant	deletion of ldhA + frdBC	1.82	[123]
Escherichia coli *	W3110, wild-type SR14, mutant	deletion of ldhA + frdBC overexpression of fhlA	1.87	[123]
Escherichia coli *	Bl-21 recombinant, mutant	deletion of hydA	3.12	[124]
Escherichia coli *	BL21(DE3] _iscR pYdbK pAF, mutant	deletion of iscR + MCS2 overexpression of YdbK + CpFdx + hydA + hydF + hydG + hydE	1.46	[125]
Clostridium paraputrificum *	M-21 pJIR751, mutant	overexpression of hydA	2.4	[126]
Clostridium acetobutylicum *	DSM 792 [pSOS], mutant	overexpression of thl promoter	1.77	[127]
Clostridium acetobutylicum *	DSM 792 [pSOShydACa], mutant	overexpression of hydA	1.81	[127]
Clostridium acetobutylicum *	DSM 792 (pSOShydACb), mutant	overexpression of hydA	1.80	[127]
Clostridium tyrobutyricum *	PAK-Em, mutant	deletion of ack	2.16	[128]
Clostridium tyrobutyricum *	PAK-Em, mutant	deletion of ack	2.61	[129]
Clostridium tyrobutyricum *	ATCC 25,755 PPTA-Em, mutant	deletion of pta	1.08	[129]
Enterobacter aerogenes *	IAM1183 A, mutant	deletion of hycA	1.20	[130]
Enterobacter aerogenes *	IAM1183 O, mutant	deletion of hybO	1.27	[130]
Enterobacter aerogenes *	IAM1183 AO, mutant	deletion of hycA + hybO	1.36	[130]
Enterobacter aerogenes *	ATCC 13048/hydA, mutant	overexpression of hydA	2.31	[131]
Enterobacter aerogenes *	IAM1183 Ea (pMCL-fdhF), mutant	overexpression of fdhF	1.16	[132]
Enterobacter aerogenes *	IAM1183 A (pMCL-fdhF), mutant	deletion of hycA overexpression of fdhF	1.19	[133]
Enterobacter aerogenes *	IAM1183 (pCOM 10-fdh1), mutant	deletion of ldh overexpression of Fdh1	1.70	[134]

* The kind of bacteria.

## Data Availability

Not applicable.

## Sample Availability

Samples of the compounds are not available from the authors.
